# Hospital Staffs and Medical Benefit

**Published:** 1913-05-03

**Authors:** 


### Hospital Staffs and Medical Benefit.

A POINT FOR HOSPITAL MANAGERS.

A hospital porter, sleeping on the hospital's premises, writes a correspondent, fell ill, and was placed in a bed in the wards. After being in the wards for a little over a week, he was sent to a convalescent home, where he stayed a fortnight at a fee of 10s. 6d. per week. His Insurance Society handed him £1 for his sickness benefit while at the home, but refused payment for the period he was warded. He was informed that had he been in his own room at the hospital instead of in a ward the sickness benefit would have been paid.

In the House of Commons on Wednesday, April 16, Mr. Boyton asked whether, under the National Insurance Act, hospital employees, being insured persons receiving attention in sickness in the wards of their hospital, are entitled to sickness benefit?

Mr. Robertson : I am advised that the answer to the question is in the affirmative.

Mr. Worthington Evans : Will the hon. gentleman ask the Commissioners to exercise their power to remove difficulties, and so prevent a porter being deprived of his benefit because he continues to live in the hospital when he is well?

Mr. Robertson : If the hon. gentleman will be good enough to give particulars they will be carefully gone into.

Mr. Worthington Evans : If the hon. gentleman will be good enough to read the supplementary question in the Official Report he will find it contains all the particulars which are required.

Mr. John Ward : Is it not a fact, even if a person does live in the hospital and has dependants, that the Approved Society is bound to pay his benefits?

Mr. Robertson : I would ask for notice of that question.

We understand that Mr. Boyton is communicating with the Secretary of the Treasury, and asking him to see that the porter gets the benefit to which he is entitled.

